# From Blank Canvas to Masterwork: Creating a Professional Practice Model at a Magnet Hospital

**DOI:** 10.1155/2016/8783594

**Published:** 2016-12-20

**Authors:** Lynda J. Dimitroff, Donna M. Tydings, Sue Nickoley, Lynn W. Nichols, Maureen E. Krenzer

**Affiliations:** ^1^Nursing Department, Nazareth College, Rochester, NY, USA; ^2^Department of Nursing Research & Evidence-Based Practice, Rochester Regional Health, Rochester, NY, USA; ^3^Rochester, NY, USA; ^4^Department of Nursing Research & Evidence-Based Practice, Rochester General Hospital, Rochester, NY, USA; ^5^Department of Clinical Education, Rochester General Hospital, Rochester, NY, USA

## Abstract

*Objective*. The purpose of this study was to engage registered nurses (RNs) in the creation of a Professional Practice Model (PPM).* Background*. PPMs are essential as the philosophical underpinnings for nursing practice. The study institution created a new PPM utilizing the voice of their RNs.* Methods*. Qualitative inquiry with focus groups was conducted to explore RNs values and beliefs about their professional practice. Constant-comparative analysis was used to code data and identify domains.* Results*. The 92 RN participants represented diverse roles and practice settings. The four domains identified were caring, knowing, navigating, and leading.* Conclusions*. Nurse leaders face the challenge of assisting nurses in articulating their practice using a common voice. In this study, nurses described their identity, their roles, and how they envisioned nursing should be practiced. The results align with the ANCC Magnet® Model, ANA standards, and important foundational and organization specific documents.

## 1. Introduction

Professional Practice Models (PPMs) are significant to the foundation of nursing practice, defining roles and leading to excellence in care delivery and positive outcomes. The American Nurses Credentialing Center (ANCC) Magnet® Recognition Program provides the framework for professional nursing practice and healthy work environments, the driving forces behind this project. Often, PPMs are imposed by administrators utilizing standard models or those that are casually generated. The goal of this study was to engage registered nurses (RNs) in the creation of a PPM.

## 2. Background and Significance

Ensuring that RNs practice according to the philosophical underpinnings of their profession is an important factor in job satisfaction [1]. Many organizations have developed PPMs with the goals of empowering the nursing workforce and improving the quality of patient care [2]. The Institute of Medicine report [3] recommends that RNs be full partners in the redesign of US healthcare. The development of a PPM is an essential contribution to the reconceptualization of the optimal role of nurses.

The Magnet Recognition Program requires organizations to show evidence of a PPM and define a PPM as follows:

… the overarching conceptual framework for nurses, nursing care, and interdisciplinary care. It is a schematic description of a system, theory, or phenomenon that depicts how nurses practice, collaborate, communicate, and develop professionally to provide the highest quality care for those served by the organization (e.g., patients, families, community) [4].

Arford and Zone-Smith [2] stated a PPM mandates that nurses decide what the activities and responsibilities of nursing are, and the competencies and educational background required to safely perform the activities. “If direct care nurses are to have control of their practice, then, fundamentally, they must decide what that practice is” [2].

Our original PPM used high level abstract concepts and language, and the nurses had difficulty connecting with it. With thoughtful consideration, a plan emerged to engage our nurses in the creation of a PPM after learning about the Middlesex PPM Project (MP) [5]. The Middlesex presentation at a Magnet Conference showcased a process that we could adopt to empower our staff in the creation of their own model.

Nurses providing leadership from the bedside to the boardroom are critical to the future of nursing care. To enable nurses to be leaders, they require a solid foundation upon which to base their practice.

## 3. Review of the Literature

Cumulative Index to Nursing and Allied Health Literature (CINAHL), Health News, Medline, and Nursing Reference Center databases were used to search the professional literature. Search terms included nursing PPMs, PPMs in health systems, PPM and Magnet, helper model, differentiated practice, relationship-based care, and group practice.

### 3.1. Professional Practice Models and Their Subsystems

Girard et al. [1] pointed out that a set of values and performance expectations to which all nurses can subscribe and that influence practice behaviors is essential to creating a culture of excellence. The American Association of Colleges of Nursing's white paper [6] described a key element of a PPM as empowered nursing clinical decision-making with authority to develop and implement nursing care orders and actions. Hoffart and Woods [7] defined a PPM as a system (structure, process, and values) that supports nurse control of the delivery of nursing care and the environment in which care is delivered. They identified five subsystems in a PPM: values, professional relationships, a patient care delivery model, a management approach, and compensation and rewards.

Hoffart and Woods [7] analyzed five existing PPMs to determine how the five PPM elements were addressed.* Professional values* included most often in these five PPMs were nurse autonomy, nurse accountability, professional development and continuing education, and high quality care. Other values included continuity in patient care, commitment to service, and critical thinking.* Professional relationships* where nurses respect each other's abilities and needs, communicate and consult effectively, and emphasize collaboration are the relationships essential for success of a PPM.

The* patient care delivery system* was the third element of PPMs. This was the structure and process by which responsibilities for patient care were assigned and work was coordinated among staff members. Historically, typical care delivery systems have included functional nursing, team nursing, total patient care, and primary nursing. Manthey [8] raised five questions to be addressed by a care delivery system. (a) “Who is responsible for making decisions about patient care? (b) How long does [sic] that person's decisions remain in effect? (c) How is the work distributed among staff members: by task or by patient? (d) How is patient care communication handled? (e) How is the whole unit managed?” [8].

Hoffart and Woods [7] discussed the fourth element of the PPM in their review (a* management or governance approach*). This element specified the structure and processes used to make decisions related to unit and organizational operations. Hospitals and nursing departments have traditionally been highly bureaucratic, but recently health care organizations have moved toward decreasing organizational layers and adopted decentralized decision-making processes. Participatory management approaches were more consistent with the professional values of autonomy and accountability than the traditional approaches. Of course, unit level governance would be facilitated with a parent organization that fosters participatory management.

The fifth subsystem of a PPM was the* compensation and rewards* system by which nurses were recognized for their contributions to patient outcomes, the organization, and the profession. Traditionally nurses have been compensated based on hours of work. However, Hoffart and Woods suggested that alternative methods such as compensation based on improved effort, increased unit productivity, and better patient outcomes were consistent with the professional value of accountability for practice.

Arford and Zone-Smith [2] stated that a PPM mandates that nurses decide what the activities and responsibilities of nursing are and the competencies and educational background required to safely perform the activities. For example, Arford and Zone-Smith [2] point out it is doubtful that direct care nurses decided their work included hunting for needed equipment or included cleaning patient rooms on off-shifts!

Erickson et al. [9] detailed the experience of the Massachusetts General Hospital in Boston on the use of the PPM. The model components they described include* values*,* philosophy*,* standards of practice*,* collaborative decision-making*,* professional development*,* patient care delivery systems*,* privileging*,* credentialing*,* and peer review research and descriptive theory models*. Some components* (values*,* collaborative decision-making*, and* patient care delivery systems)* parallel those discussed by Hoffart and Woods.

### 3.2. Potential Benefits of PPMs

Ingersoll et al. [10] pointed out that linking nursing PPM components (and performance evaluation criteria) with an organization's mission, vision, and values statements assured that the organization's values, beliefs, and intentions were evident in daily work life.

A PPM was developed and implemented in London, Ontario's London Health Science's Centre's renal program [11]. The authors conducted a study in one unit to examine the impact of the PPM on nurses' perceptions of empowerment, characteristics of practice environments, and nursing outcomes. Kanter's [12] theory of empowerment provided the framework for the study. There was a significant (*p* = .005) improvement post-PPM implementation in the nursing foundations for quality of care subscale (one of five subscales) of the Nursing Worklife Index-Practice Environment Scale (NWI-PES). Nurses' perceptions of empowerment were measured by the Conditions of Work Effectiveness II (CWEQ-II) Questionnaires. Scale scores on the informal power subscale were significantly increased after implementation (*p* = .016).

Hastings [13] reported on the Professional Practice Partnership model at the University of Maryland Medical System which was implemented across all 52 units. Evaluation studies identified significant unit variability in ways the model evolved and in staff nurse perceptions of effectiveness. For the secondary analysis, Hastings created two subsamples of the original sample, one with staff from 11 critical care units and one including staff from 15 general care units. Five predictors of critical care nurses' general job satisfaction (ability to give high quality care, perception of peer support, care delivery system effectiveness, involvement in decision-making, and satisfaction with scheduling) explained 48% of the variance in general job satisfaction. For acute-care nurses' job satisfaction, four predictors were the same and care delivery system effectiveness was replaced by nurse-patient ratio.

Critical care nurses, in contrast to general care nurses, felt more positively about peer support and decision-making involvement, key aspects of the practice model, and were more satisfied with the amount of control and responsibility they had in their roles. Hastings considered that these factors may support the existence of tight collaborative relationships and mutual trust, which are important in building a successful model.

Needleman et al. [14] were responsible for an evaluation of the Transforming Care at the Bedside (TCAB) program. The program evaluation included focus on data collected via semistructured interviews of staff involved, data from semistructured telephone interviews at the end of year two, and innovations reported during the two years. The Robert Wood Johnson Foundation (RWJF) [15] evaluation team reported the following:

- “The small number of participating hospitals limited the ability of the evaluators to say whether the program improved patient care.
- The program was successful in engaging frontline staff in developing, testing, and implementing changes to improve processes on nursing units.
- The work of testing and evaluating innovations spread beyond the original nursing units participating in the program to other parts of the participating hospitals” [15].

Upenieks et al. [16] described the number and type of innovations tested on 16 units in 13 hospitals involved in the Transforming Care at the Bedside (TCAB) program. The process included encouraging frontline staff members to participate in brainstorming sessions to identify unit priorities and decide on the types of innovations that they felt would enhance their work conditions and produce safer, more reliable patient-centered care. They discussed improving vitality (impact of the work environment on the nurses) and value/lean to increase effectiveness, promote continuous workflow, and reduce waste and low-value work. Across the 16 units a total of 426 innovations were tested.

Nurse managers and staff from seven units self-reported an increase in vitality in both years of the study period; five units reported an increase in vitality in one year only; and four units reported no improvement in vitality in either year. On units where vitality increased, the predominant themes identified by nurse leaders included empowerment, increased accountability, and a sense of ownership of the unit. One leader said, “Vitality is high for several reasons—brainstorming change efforts are generated from the staff level to the leadership level, staff are able to speak to the goals of the project, and staff are praised for quick wins” [16]. Units that reported no change in vitality conducted fewer tests of innovations than did the other units. This link of involvement in testing and implementing changes in care on their units to vitality was supported by previous research on Magnet hospitals [16] that demonstrated a relationship between the level of nurse job satisfaction and access to empowering factors in the workplace and the ability to exercise judgment and implement changes related to their work environment.

Vitality might be equated to engagement. Freeney and Tiernan [17] contend that there was no clear agreed upon definition of engagement and presented a definition of work engagement as “a persistent, positive, affective-motivational state of fulfillment in employees that is characterized by vigor, dedication, and absorption.” Maslach and Leiter [18] identified six areas of organizational life that contribute either to engagement or its opposite: burnout. To facilitate engagement, nurses want to perceive the workload as manageable, to have control over one's work by being part of the decision-making process, to be recognized and rewarded for their work, to have a positive connection and sense of community with others in the workplace, to feel they were treated with fairness, and to find their work to be in line with their values. In their qualitative study with semistructured focus groups with 20 nurses, Freeney and Tiernan [17] reported that the themes they identified strikingly mapped onto the Maslach and Leiter burnout and engagement model. They concluded there was an urgent need to design and evaluate an intervention program that focuses on fostering engagement.

Wong et al. [19] studied the costs of nursing care in eight nursing units at the Johns Hopkins Hospital that had adopted a PPM and compared costs with eight nursing units that had not. They described the PPM at the hospital. Nurses of a PPM unit enter into an agreement with the hospital and took responsibility as a group to provide 24-hour nursing care in the unit for one year. They managed themselves, received salaries rather than hourly wages, and shared in a salary incentive based on the unit's performance. Nursing salaries in PPM units were upgraded 10% over non-PPM nurses to compensate for foregone earnings from shift work, overtime, or holiday differentials. Labor cost savings at the unit level determined the dollar amount of the incentive and the share of the cost savings for nurses was negotiated annually with the hospital. The nurses determined the share for each individual within the unit.

Results varied. For ORs, the PPM units were over 40% more costly than non-PPM units in terms of cost per OR day. The increased costs related to the fact that the PPM OR units used more time from RNs than did the non-PPM OR units. On the other hand, for inpatient units, cost per patient day was similar. PPM inpatient units used fewer temporary personnel and nursing aides (i.e., unlicensed assistive personnel) but similar hours of RNs, resulting in similar RN costs but lower total nursing costs. Wong et al. [19] stated since turnover is reduced on PPM units, the cost of recruiting and training new nurses is lower on PPM units.

### 3.3. PPM That Focuses on Caring

The relationship-based care (RBC) model was the subject of a text edited by Kolurotis [20]. One of Hoffart and Woods' elements of the PPM addresses professional values, including a focus on high quality care. The Fetzer Institute and the Pew Health Professions Commission Task Force [21] have identified the concept of relationship-centered care as key to the delivery of high quality health care.

The RBC model evolved from 25 years of experience of the organization entitled Creative Health Care Management, Inc. [20]. Selected conclusions that the organization has drawn include the following:

1. Patients and families define “caring and healing environments” as those in which they are actively involved in their own care … and where they have established an individualized relationship with physicians, nurses, and other care providers.
2. The nurse-patient relationship is the foundation of excellent care delivery, and nurse accountability for a therapeutic relationship with a patient and the patient's family is essential to achieving quality outcomes.
3. Patient involvement and confidence in their care are increased by positive relationships with their care providers.
4. Patient safety is more effectively safeguarded when … a nurse knows the patient, the patient's family, and what matters most to all of them.
5. How an organization's leaders regard the value of the nurse-patient/family relationship within the context of a collaborative team effort determines how work is structured and what is prioritized.
6. Organizations with caring and healing environments and a focus on relationships have higher patient, staff, and physician satisfaction and higher productivity [20].

Kolurotis [20] stated the RBC model provides the philosophical foundation and practical infrastructure to achieve organization-wide transformation in the way care and service are provided to patients and their families. The six dimensions needed for implementation of RBC were leadership, teamwork, professional nursing practice, patient care delivery, resource driven practice, and outcomes measurement.

Kolurotis [20] identified six roles of nursing practice:

1. sentry (one who watches over and protects),
2. healer (one who cares for another's body, mind, and spirit; one who helps others improve their level of health),
3. guide (someone who leads or directs another's way through unfamiliar circumstances; one who possesses intimate knowledge of the way),
4. teacher (one who imparts knowledge; someone who helps another learn a skill),
5. collaborator (one who works cooperatively with others to achieve a common purpose),
6. leader (someone who has the authority to act on behalf of others and possesses the capacity to effect change and influence direction).

Slatyer et al. [22] completed a systematic review identifying the key components of a PPM synthesized from a qualitative analysis of 51 articles describing 38 models. The authors concluded that the main elements common to all PPMs were (1) having a theoretical foundation (e.g., nursing concepts such as shared governance, relationship-based care, or forces of magnetism), organizational core values, or external theory and (2) six components (leadership, nurses' independent and collaborative practice, environment, nurse development and recognition, research/innovation, and patient outcomes).

## 4. Purpose and Research Question

The purpose of this study was to engage RNs in the creation of a PPM. The research question for this study was the following: how do registered nurses in acute-care hospitals conceptualize their professional practice?

## 5. Methods

The method for this study was naturalistic inquiry using a descriptive qualitative tradition. “Qualitative descriptive is especially amenable to obtaining straight and largely unadorned … answers to questions of special relevance to practitioners …” [23]. One hallmark of qualitative research is to make meaning through discovery.

Discovery is defined as the presentation … of new perspectives on or information about the human phenomenon under study. New perspectives or information may be revealed, for example, in verbatim accounts that portray the experience under study for the first time or with previously uncaptured richness, or in a theoretical or interpretive framing of the phenomenon that sheds light on how it came to be and what it is like [24].

Focus groups (FGs) have precedence in data collection [1] to develop PPMs and allowed for our nurses' voices to be heard. The study sought to understand what it means to be a RN.

## 6. Participants and Recruitment

RNs were contacted to participate in this study through e-mail, unit huddle announcements, and unit posters. In addition, announcements regarding the study were made at meetings including care managers, directors, education council, nurse council, nurse managers, pain resource nurses, and town meeting.

Nurses who used email were sent an electronic meeting invitation and asked to accept or decline the invitation. This invitation assisted with the scheduling of FG facilitators and assistant facilitators. Direct care nurses who were interested in participating in this study were asked to contact the hospital's registration telephone line to register for a FG. The staff at the registration center were made aware that the registrations for this FG were related to a research study so it was important to maintain the confidentiality of those who called to register.

To be eligible to participate in the study participants met the following inclusion criteria: (1) being a registered nurse, (2) working as a RN in the acute-care facility, (3) a willingness to participate in the study, and (4) being at least 18 years old. Exclusion criteria included any RN who worked in an affiliate institution or medical practice.

## 7. Study Setting

The study was conducted at a 528-bed acute-care urban teaching Magnet designated hospital. This hospital has been serving the community of greater Rochester, NY, and beyond for the health care needs of residents of western and central NY state for more than 150 years.

## 8. Data Collection

Institutional review board approval was obtained prior to data collection. All researchers completed the National Institute of Health's Human Subjects Participant Protection program prior to data collection. Data were collected through FGs using open-ended questions adapted from the MP as well as a demographic form which was given to the participants for completion once the FG ended. Demographic data collected included gender, highest degree held in nursing, highest degree held outside of nursing, employment status, number of years as a RN and number of years on present unit, current role, and area of practice. FG open-ended questions were as follows:

1. When did you first really feel like a nurse?
  What was the defining moment?
  Tell me a story about the first time you felt like a nurse.
2. Imagine someone you love very much is a patient. What kind of nurse would you like to take care of this person?
3. Describe an ideal day to work at this hospital.
  What are the characteristics of an ideal day?
  Describe a perfect day to be a nurse.
4. A reporter asks, “How is a nurse's job different from other caregivers in the hospital?”
  What would you tell them?
  How would you respond?
5. As a RN, what are you most proud of?
6. Do you have any questions for us?

There were nine initial FGs with 66 RNs representing all settings and levels of nursing practice. Focus groups were conducted by the PI or her designee (trained to conduct FGs), with the assistance of a trained assistant facilitator. Focus groups lasted one hour, were audio-recorded, conducted until data saturation was reached, and transcribed verbatim.

Focus groups were conducted at the acute-care facility where the study took place and were scheduled at a time and place that was convenient for both the participants and facilitators. The goal was to use regularly scheduled meetings to conduct FGs when possible. For FGs that were scheduled during regular meetings, an announcement was made at the meeting prior to the scheduled FG and nurses were told that if they did not want to participate in the study they should not attend the following meeting.

At the beginning of each FG, a script was read detailing the purpose of the study, how data were to be collected and analyzed, and steps taken to ensure confidentiality. During this time, it was explained that participation in the FG implied consent.

The proposed times to hold FGs for direct care nurses included 0400–0500, 0730–0830, 1100–1200, 1400–1500, 2000–2100, and 2200–2300. Two consecutive weekends were utilized as many direct care nurses work every other weekend.

All information from the FGs was confidential as no participant names were used during analysis. All identifying information was removed from the transcripts to ensure confidentiality. Member checks to confirm the initial categories and domains were conducted with 26 RNs through two verification FGs. FG audio-recordings were transcribed by a paid consultant who was familiar with research transcriptions.

## 9. Data Analysis

Consistent with the ongoing process of interpretation in qualitative research, the transcripts were read, coded, and reread by all members of the research team. Data were analyzed by all members of the research team allowing for auditing categories thus ensuring the dependability and confirmability components of trustworthiness. Credibility was ensured through member checks to make sure that what the participants said was accurately documented. Transferability was ensured through verbatim transcriptions, the use of direct quotes to substantiate the themes, the use of a purposeful sample, creating detailed methods and data collection sections for accurate replicability of the study, and the timely conversion of all transcripts for data analysis. Using constant-comparative analysis, the common categories identified were classified into domains and used to answer the research question. Direct quotes from the transcripts were used to substantiate domains.

The verification FGs validated four domains and related subdomains. The participants provided minor suggestions and overwhelmingly validated the model as representative of their nursing practice.

## 10. Results

Demographic data were analyzed using descriptive statistics (Table 1). The 66 participants represented a variety of roles and practice areas 43% (*n* = 29) direct care, 22.7% (*n* = 15) clinical leadership, and 18% (*n* = 12) management. Fifty-four percent (*n* = 36) were baccalaureate prepared, 18% (*n* = 12) were master's prepared, and 48.5% (*n* = 32) had more than 21 years of nursing experience.

During data analysis,* caring*,* knowing, navigating, and leading* were identified as the four domains of the PPM (Table 2). Each domain, fully detailed by subdomains and definitions, added depth and dimension essential to the full understanding of this model. Subdomain and definitions were as follows:

*Caring*

- *A holistic approach* is knowing the patient as a person and human being, tending to the body, mind, and soul. It is acknowledging the importance of the patient's family, that the family is a part of the whole. A holistic approach is being empathetic and putting oneself in the patient's shoes.
- *Affirmation* is patients and families expressing their sincerest thanks and gratitude to the nurse.
- *Connection* is being in the moment with patients and families, beyond what is regarded as safe care. It is time spent with patients unrelated to tasks and doing the extra little things.
- *Time* is identified as the nurse taking time to be present with patients.
- *Trust* is patients and families depending on the nurse to always be there. It is safeguarding and respecting the confidential and personal information shared by patients and families.

*Knowing*

- “*Big picture*” is the nurse seeing the comprehensive clinical picture and synthesizing the information to take action.
- *Competence* is being clinically skilled and capable of implementing best practice.
- *Critical thinking* is the application of knowledge to assess, connect the dots, and problem solve. It is the ability to express the rationale for nursing action and to anticipate changes.
- *Intuition* is a gut feeling of knowing that something is amiss or that an adverse event may occur.
- *Lifelong learning* is a nurse's personal commitment to continuing education and professional development. It includes achieving and maintaining certification, obtaining advanced degrees, attendance at conferences, and participation in continuing education.
- *Nursing as a profession* is the art and science of applying knowledge and clinical experience to impact patients. Nursing as a profession is about the development and mentoring of new nurses to promote autonomy and the use of evidence-based practice. Nurses share a unique bond with each other.

*Navigating*

- *Advocacy* is the nurse's persistent action to give patients a voice and to act on their behalf.
- *Communication* is the exchange of information between nurses, team members, patients, and families. Communication involves patient updates, rounding, keeping patients informed, and providing explanations.
- *Hub* is the nurse being the pivotal point and the center of patient care. The nurse serves as the link between the patient, family, and team to coordinate and integrate patient care.
- “*Making a difference*” is the nurse impacting patients and families to produce significant outcomes and to create imprints with long-lasting effects on their lives.
- “*Master of all trades*” is the nurse having the ultimate responsibility and mastery of many roles related to patient care. Nurses orchestrate the day-to-day, “behind the scenes” activities often invisible to others.
- *Support* is having what is needed to provide for patients and team members. Support includes proper equipment and resources, adequate staff and skill mix, and the emotional care for each other in the day-to-day operation of the unit.
- *Teamwork* is working together in synergy with team members. It is having a positive “can do” attitude, being compatible with others and ultimately getting the job done.
- *Time* is the nurse having adequate time to complete tasks, and ensuring meals and breaks occur regularly.

*Leading*

- *Affirmation* is the expression of sincerest thanks, gratitude, and recognition for the nurse's contributions from colleagues, supervisors, physicians, and community members.
- *Global vision* is the nurse seeing the whole picture, visualizing the overall system, and synthesizing the information to take action.
- “*Making a difference*” is the nurse impacting systems and communities to produce significant outcomes and to create imprints with long-lasting effects.
- *Nurses as professionals* portray a positive image which includes a professional appearance, being on time, conducing themselves with integrity, and utilizing effective communication skills.
- *Respect* is consideration and courtesy to and from patients, nursing colleagues, and others. It includes listening and treating others with dignity.
- *Support* is fostering professional development by administrators and having what is needed to take care of patients and team members. Support includes proper equipment and resources, adequate staff and skill mix, and emotional care for each other.

**Figure 1:**
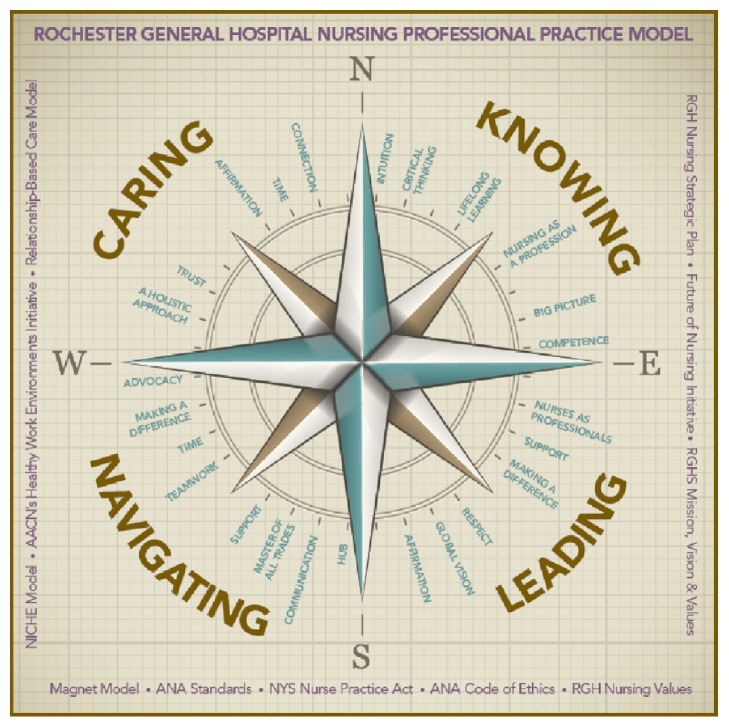
Professional Practice Model.

The domains and subdomains were designed into an illustration to depict their interactions (Figure 1). 

*Domain I: Caring*. Caring was defined as the essence of nursing in an affective (emotional) demonstration of commitment to patients and families. One participant recalled a caring moment provided to her dad by a nursing colleague.

what this nurse provided my family member was caring. One day I walked in … and she (the RN) was sitting on the bed holding my father's hand. … she was talking about end-of-life decision making. … she was a bedside nurse… she said, “I try and do this with my hospice patients … on a Sunday morning when it's quiet and no family is there. That's my time with my patient.” … I thought, that's what I was looking for (FG#1).

A holistic approach, connection, time, and trust emerged as subdomains of caring and were acknowledged through affirmation from patients and families. One nurse described this affirmation as “… one of those days when you say goodbye to your patient and they say ‘thank you so much. Are you going to be my nurse tomorrow? I really want you to be my nurse tomorrow'” (FG#8).

The participants described the importance of understanding the patient as a person and human being, taking the time to be in the moment with them, and tending to their body, mind, and soul. “… not just be present to tell me what the monitor's doing … They (RNs) have to listen and … deliver human holistic care” (FG#9). One participant expressed “It's the look, it's the touch, it's just being there, being with the patient. Not just dealing with the tube … it's actually … looking into their eyes, holding their hands, gentle touch. Those kinds of things make all the difference in the world to the patient and family” (FG#1).

*Domain II: Knowing*. Knowing was defined as the art and science of nursing, an essential attribute to the success of nurses and the safe delivery of patient care. It was the translation of embodied knowledge into evidence-based clinical decisions, actions, and scholarship. One participant had this to say about when she first felt like a real nurse. “There was an endorphin high … I felt that everything I'd worked towards was finally coming together. I felt more confident in myself … I could make some decisions without … asking every time … I felt great for the patient that I made the right decision for her” (FG#8).

The subdomains comprising knowing were competence, critical thinking, intuition, life-long learning, nursing as a profession, and “seeing the big picture.” One participant's description illustrates several subdomains.

Even though I believe that all members of the patient care team are equally important and that includes housekeeping, laundry, food … services … we (RNs) have to use evidence-based practice and concrete knowledge of what's going on with our patients and what we think is best for our patients as we advocate for them (FG#9).

Another participant simply stated, “… nursing is an art and a science. You have all the science you learned and the art of compassion and putting it all together” (FG#5).

In defining intuition, one nurse stated, “… It's hard to explain … It's that looking at the patient and just knowing … There's something going on with them and I need to get someone here to see them” (FG#1).

*Domain III: Navigating*. Navigating characterized the nurse's role on the team guiding patients and team members through the complexities of the health care experience. This domain encompassed the RN having the ultimate responsibility and accountability for establishing the link between all health care team members to navigate on behalf of patients. The subdomains for navigating included advocacy, communication, hub, “making a difference,” “master of all trades,” support, teamwork, and time.

Participants described the nurses' role as pivotal to patient care. They repeatedly conveyed the nurse as the center and how critical the nurse is in advancing the patient along the continuum of care. “We're the hub that makes the wheel turn. If it weren't for nursing that wheel wouldn't turn, and if the wheel doesn't turn the patient's not going anywhere” (FG#2).

The participants acknowledged the contributions of the entire healthcare team in achieving superior outcomes. The RNs communicated that team function was highly dependent on adequate support, time, and resources and the nurse's facilitation of effective communication processes among team members to keep patients informed. One participant put it this way:

… staffing, staffing, staffing. … you could have a million people on but if you don't have them functioning as a team and having the right attitude and … really wanting to give care, it doesn't matter how much staff you have on. It's all who's partaking in the care (FG#3).

The “master of all trades” was a recurrent phrase used by participants to impart the nurses' obligations. One participant described it aptly:

I think that you're beyond doing even their care. … you're advocating for the patient and … moving the furniture, talking to the physician,… talking to the family,… calling when something is broken,… making sure they get their meal trays. Everything ultimately is the responsibility of the nurse, bottom line. You have a physician, you've got techs, you've got a million people to make this hospital run, but bottom line … everything seems to be the responsibility of the nurse (FG#3).

The participants expressed the importance of advocacy as an integral component of navigating. They recognized that patients are often unable to navigate on their own and it was the nurse who provided this essential service. The participants could not imagine nursing without advocacy and expressed their passion for serving in this role.

I'm proud to be the patient's voice when they don't have a voice and they don't understand what's going on with their care, when they don't have a family member to help them. I think that's one of the things I'm most proud of, is being their voice (FG#3).

*Domain IV: Leading*. Leading, the fourth and final domain, was defined as nurses organizing people and processes, charting new directions, and having a vast sphere of influence on patients, families, and the nursing profession within the organization and community. Leading encompassed affirmation, the nurses' global vision, the ability to “make a difference,” nurses as professionals, respect, and support through the fostering of development by administrators. One nurse described the long-lasting imprint that nurses make on the lives of others,

I think nurses make an impact … every single day. In other professions, you can make an impact every once in awhile [sic], but we come to work and everyday [sic] we make an impact … The way that we do our jobs can define the rest of somebody's life … and not just them, but their family, their children (FG#6).

Other subdomains of leading were affirmation and respect which were illustrated in what this nurse had to say about a physician colleague:

… I remember years ago that there was a new resident … I remember talking to this intern and said “if you want to make [it] I'll tell you some words of wisdom. Always listen to the nurse. If I call you and say you've got to come up and see a patient, realize that these nurses are with the patient 8–10 hours a day, so if they call you and say there's something wrong, come up and see them. If you want to be good, if you want things to go smoothly, I recommend you listen to the nurse. Take their words of wisdom. If they have any suggestions, listen to them because they have been around the block a few times; this isn't their first rodeo” … he looked at me and said, “thank you” and then he helped me make a bed (FG#6).

The nurses repeatedly provided examples of how the domains and subdomains applied to their professional practice and discussed the value of the results. They said, “wow, this summarizes who we are, and what we do!”, and “… it puts it into words; you don't think about doing each of these things, but when you read it, it's like, ‘Oh yeah, we do that …'” One telling quote was, “It's interesting because when you get up and go to work in the morning, you don't realize this is what you do every day, all day long…!” The nurses took great pride in the fact that they created their PPM through participation in nursing research.

## 11. Discussion

The significance of the results was most importantly that this PPM gave voice to our nurses and our profession. The description of the nurses' practice contributed to the creation of our PPM. While these descriptions provide the essence or “heart and soul” of our PPM, key models and important documents provide its foundation. Our PPM provides solid underpinnings for practice as supported by key models and foundational documents: legal and regulatory aspects (ANA Code of Ethics for Nurses and Nursing Scope [26] and Standards of Practice [27]) and nursing standards of excellence (AACN's healthy work environments [28] and the Future of Nursing Initiative [3] and Magnet and NICHE models), nursing organizational specific values (mission, vision, values, and strategic plan), and the Kolurotis Relationship-Based Care Delivery model [20]. These documents serve as the anchor and come alive as RNs operationalize the PPM in daily practice.

Following data analysis, a number of initiatives were implemented to continue nurses' engagement in the PPM. Initially, the PPM research team provided comprehensive education on the model via live interactive sessions for all nursing staff. Concurrently, framed pictures of the PPM were prominently displayed on each nursing unit. In addition, each unit's nurse sensitive indicator data were posted on bulletin boards organized by the PPM domains. This public display enabled both hospital staff and visitors to connect patient outcomes to the PPM. The PPM was also incorporated into the annual self- and peer performance evaluations and into nursing orientation. More recently, a story template was developed to capture narratives of nursing process improvement activities. This worksheet provides guidance to nurses embarking on improvement initiatives and enables them to measure the resulting impact.

When comparing our model to other published nursing PPMs, similar components were identified despite the varying language used [9, 22, 29]. A review of the literature by Slatyer et al. [22] identified six key concepts that were present in all of the studies reviewed. The concepts were leadership; nurses' independent and collaborative practice; environment; nurse development and recognition; research/innovation; and patient outcomes. While the terminology differed, our model's concepts parallelled those in the literature such as leading, teamwork, respect and support, affirmation, knowing, and making a difference.

Several strengths of our PPM were identified. The project was conducted as a formal study with the method section written in an easily replicatable format and the qualitative method captured the profound words of our RNs. The uniqueness of our PPM is in the integration (sum is greater than the individual parts), balance (each domain is equally important), interaction of domains and subdomains, and the depth of each domain further described by the subdomains. These subdomains enhance the richness of each domain using terms adding clarity and meaning. Subdomains exist in few other PPMs.

Our model describes how nurses practice at the bedside and beyond. The model is dynamic and has a great deal of flexibility, allowing applicability to different roles and practice settings. Within each domain nurses can grow from novice to expert and transition at different rates. This PPM speaks to how nurses practice, collaborate, communicate, and develop professionally, key components in the Magnet definition of a PPM.

Developmental levels through which hospitals progress as they evolve throughout their ongoing Magnet journey were identified by Wolf et al. [25]. The authors describe four levels useful in measuring the degree of enculturation of an organization's PPM ranging from reactive to high performing (Table 3).

Prior to embarking on this study, the participating organization was at the reactive level in relation to its PPM. Nursing staff lacked knowledge of the organization's PPM and were unable to describe it let alone use it to guide nursing practice. Following the completion of this study, a number of initiatives were implemented to operationalize the PPM and bring it to life for the nurses. Initiatives implemented to operationalize the PPM included the following:

- providing comprehensive education on the model to all nursing staff,
- framing PPM pictures for display on all nursing unit,
- posting information on unit bulletin boards organized by PPM domains to publicly display nurse sensitive indicator data,
- developing a story template linking the PPM domains to proactively capture stories of the impact nurses make on outcomes,
- discussing at unit huddles,
- incorporating the PPM into annual self- and peer evaluations, nursing orientation, and preceptor class.

Because of these initiatives, during our 2014 Magnet redesignation site visit, RNs could speak to the PPM, define its domains, and give examples of its use in their practice. This scholarly work has allowed us to visualize the evolution of the PPM from a mere existence in the organization to enculturation of the model amongst the nursing staff.

## 12. Limitations

One limitation of this study was that participants were nurses from a single hospital. In addition, the analysis and conclusions in this article may not have been what others would have developed or extrapolated from the data.

## 13. Conclusions

This study resulted in the creation of a dynamic PPM for RNs by RNs. Utilizing their voices we created a PPM that provides a foundation on which to practice, leads us on the ever changing journey of our profession, and offers a vision of how we want to practice. This study adds to the literature providing an example of what professional practice looks like and how it is articulated. Currently there is little evidence of direct care RNs developing a PPM; therefore, this study was valuable in filling this gap. Our PPM demonstrates the depth and flexibility of nursing practice at the bedside and beyond. As one nurse stated “… it … summarizes who nurses are and what nurses do … all-encompassing from A to Z – everything” (FG#1). This scholarly work continues as the researchers integrate the PPM into daily practice and evaluate its impact on outcomes. The study is being replicated at other hospitals within our system.

## 14. Implications for Nurse Leaders

Nurse leaders face the challenge of assisting nurses in articulating their practice using a common voice. For Magnet designated hospitals and those on the journey, it is the PPM that can and will provide that voice. Application of rigorous research methods to create this PPM constituted an innovative strategy to advance the science of nursing in a replicatable format that nurse leaders can use to develop their own PPM.

The use of words that have meaning for nurses, the easily recognized compass image, and the depicted foundation for quality nursing practice can be translated to other institutions. The alignment, synthesis, and integration of ideal and day-to-day practice, the context of the work environment, and the foundational documents provide nurses with a steady source of guidance in the ever complex world of healthcare. The PPM serves as our “Magnetic” north, guiding our journey and providing us with the ability to travel where we desire to go even when the course is unclear.

## Figures and Tables

**Table:** (a) Registered nurse roles

Current role	*n*	%
Direct care positions		
Clinical leader	7	10.6
Clinical resource nurse	6	9.1
Direct care nurse	16	24.2
Clinical leadership positions		
Care manager	9	13.6
Clinical nurse specialist	6	9.1
Management positions		
Director of nursing	3	4.5
Nurse manager	8	12.0
Senior leader	1	1.5
Other responses		
Other	9	13.6
No response	1	1.5

Total	66	100

**Table:** (b) Areas of practice

Areas of practice	*n*	%
Administration	1	1.5
Clinical education	4	6.1
Critical care nursing	3	4.5
Emergency nursing	2	3.0
Medical nursing	9	13.6
Outpatient nursing	4	6.1
Pediatric nursing	1	1.5
Perioperative nursing	5	7.6
Surgical nursing	13	19.7
Women's health, obstetrics, neonatal	1	1.5
Other	23	34.8

Total	66	100

**Table:** (c) Years as a registered nurse

Years	*n*	%
Less than one year–1 year	3	4.5
2–5 years	3	4.5
6–10 years	8	12.1
11–15 years	14	21.2
16–20 years	6	9.1
21–25 + years	21	48.5

Total	66	100

**Table:** (d) Registered nurse degrees

Diploma		Associate's degree		Bachelor of science		Master of science		Total	
*n*	%	*n*	%	*n*	%	*n*	%	*n*	%
4	6.1	14	21.2	36	54.5	12	18.2	66	100

**Table 2:** PPM domains and subdomains.

Domain	Domain definition	Subdomains
Caring	Caring is the essence of nursing in an affective (emotional) demonstration of commitment to patients and families	(i) A holistic approach (ii) Caring is acknowledged through affirmation (iii) Connection (iv) Time (v) Trust

Knowing	Knowing was the art and science of nursing, an essential attribute to the success of nurses and the safe delivery of patient care. Knowing is the translation of embodied knowledge into evidence-based clinical decisions, actions, and scholarship	(i) “Seeing the big picture” (ii) Competence (iii) Critical thinking (iv) Intuition (v) Life-long learning (vi) Nursing as a profession

Navigating	Navigating characterizes the nurse's role on the team guiding patients and team members through the complexities of the health care experience. It is the nurse having the ultimate responsibility and accountability for establishing the link between all health care team members to navigate on behalf of patients. Team function is highly dependent on adequate support, time, and resources and the nurse's facilitation of effective communication processes among team members to keep patients informed	(i) Advocacy (ii) Communication (iii) Being the hub (iv) “Making a difference” (v) “Master of all trades” (vi) Support (vii) Teamwork (viii) Time

Leading	Leading is organizing people and processes. Organizational and community leadership is charting new directions and having a vast sphere of influence on patients, families, and the nursing profession	(i) Affirmation (ii) Global vision (iii) “Making a difference” (iv) Nurses as professionals (v) Respect (vi) Support

**Table 3:** Magnet developmental levels.

Reactive	“PPM may exist, but nurses do not see how it guides or relates to practice”
Responsive	“Some general understanding or acknowledgement of a PPM, but not enculturated by staff”
Proactive	“PPM is fully enculturated and serves as a ‘roadmap' for potentially other disciplines and the organization to guide practice”
High performing	“PPM is ‘owned' by staff to drive the work of nursing. The PPM impacts all aspects of practice and is leveraged to accomplish goals”

Wolf et al. [25].
